# Pharmacokinetics and Pharmacodynamics of Fungal Defensin NZX Against *Staphylococcus aureus*-Induced Mouse Peritonitis Model

**DOI:** 10.3389/fmicb.2022.865774

**Published:** 2022-06-01

**Authors:** Xueling Zheng, Na Yang, Ruoyu Mao, Ya Hao, Da Teng, Jianhua Wang

**Affiliations:** ^1^Gene Engineering Laboratory, Feed Research Institute, Chinese Academy of Agricultural Sciences, Beijing, China; ^2^Innovative Team of Antimicrobial Peptides and Alternatives to Antibiotics, Feed Research Institute, Chinese Academy of Agricultural Sciences, Beijing, China; ^3^Key Laboratory of Feed Biotechnology, Ministry of Agriculture and Rural Affairs, Beijing, China

**Keywords:** fungal defensin NZX, *Staphylococcus aureus*, intracellular bactericidal activity, pharmacokinetics, pharmacodynamics, mouse peritonitis model

## Abstract

*Staphylococcus aureus* (*S. aureus*) is one of the most common pathogenic bacteria responsible for causing a life-threatening peritonitis disease. NZX, as a variant of fungal defensin plectasin, displayed potent antibacterial activity against *S. aureus*. In this study, the antibacterial and resistance characteristics, pharmacokinetics, and pharmacodynamics of NZX against the *S. aureus* E48 and *S. aureus* E48-induced mouse peritonitis model were studied, respectively. NZX exhibited a more rapid killing activity to *S. aureus* (minimal inhibitory concentration, 1 μg/ml) compared with linezolid, ampicillin and daptomycin, and serial passaging of *S. aureus* E48 for 30 days at 1/2 × MIC, NZX had a lower risk of resistance compared with ampicillin and daptomycin. Also, it displayed a high biocompatibility and tolerance to physiological salt, serum environment, and phagolysosome proteinase environment, except for acid environment in phagolysosome. The murine serum protein-binding rate of NZX was 89.25% measured by ultrafiltration method. Based on the free NZX concentration in serum after tail vein administration, the main pharmacokinetic parameters for T_1/2_, C_max_, V_d_, MRT, and AUC ranged from 0.32 to 0.45 h, 2.85 to 20.55 μg/ml, 1469.10 to 2073.90 ml/kg, 0.32 to 0.56 h, and 1.11 to 8.89 μg.h/ml, respectively. Additionally, the *in vivo* pharmacodynamics against *S. aureus* demonstrated that NZX administrated two times by tail vein at 20 mg/kg could rescue all infected mice in the lethal mouse peritonitis model. And NZX treatment (20 mg/kg) significantly reduced CFU counts in the liver, lung, and spleen, especially for intracellular bacteria in the peritoneal fluid, which were similar or superior to those of daptomycin. *In vivo* efficacies of NZX against total bacteria and intracellular bacteria were significantly correlated with three PK/PD indices of ƒAUC/MIC, ƒC_max_/MIC, and ƒT% > MIC analyzed by a sigmoid maximum-effect model. These results showed that NZX may be a potential candidate for treating peritonitis disease caused by intracellular *S. aureus*.

## Introduction

Peritonitis is a life-threatening infectious complication encountered in patients, pets, and livestock, mainly from the process of gastrointestinal perforation, intra-abdominal surgery, and peritoneal dialysis, being as some prerequisites for microbes to enter the abdominal cavity (Broughton et al., 2010; Pörner et al., 2021). *Staphylococcus aureus* (*S. aureus*) is one of the most common pathogens associated with peritonitis (Salzer, 2018; Marciano et al., 2019; Szeto et al., 2021). Although antibiotic therapy is a common strategy for eliminating bacterial infection, peritonitis caused by *S. aureus* remains a major complication of peritoneal dialysis with the characteristics of persistence and recurrence (Marciano et al., 2019; Alonso et al., 2021; Szeto et al., 2021). One of the major causes of recurrent peritonitis is from the emergence of antibacterial resistance during antibiotic treatment, especially from methicillin-resistant and multiple resistant strains (Li et al., 2020a; Camargo et al., 2021). In addition, *S. aureus* can invade and replicate in various host cells, complicating the usage of antibiotics (Zhou et al., 2018; Wang et al., 2021); therapeutic effects were impeded by poor penetration or inferior intracellular stability. Therefore, there is an urgent need to develop new intracellular antibacterial drugs with low resistance and high cellular permeability for the treatment or prevention of *S. aureus*-related peritonitis diseases.

Antimicrobial peptides (AMPs) possess the advantages of rapid killing, high specificity, and low resistance to pathogens compared to other small molecule drugs (Liu et al., 2021; Moretta et al., 2021; Rima et al., 2021). Additionally, AMPs proved to be potent in inhibiting or eradicating intracellular organisms (Brinch et al., 2009; Wang et al., 2018, 2019; Li et al., 2020b, 2021). Also, the iron triangle theory on health protection from AMPs, antibiotics, and vaccines for maintaining a reasonable equilibrium among pathogens, antimicrobials, and drug resistances has been recently put forward (Zheng et al., 2021; Hao et al., 2022). Given those merits, AMPs have been considered as promising and new therapeutic anti-infection candidates, and should exert more roles in health practices of humans and animals. However, to date, very few new drug applications of AMPs have successfully passed official approval and entered into clinical application despite a lot of AMP-related patents and papers achieved (Mercer and O'Neil, 2013; Costa et al., 2019). One main obstacle is the instability of AMPs due to their fast metabolism in host body; this is a double-edged sword in terms of methodology in evaluation detection and reality in safety monitoring; on one hand, AMPs are hardly accumulated in the host body, thereby reducing drug residues and resistance as their merits (Henninot et al., 2018; Costa et al., 2019); on another hand, their metabolites or small segments in distribution details in a biological matrix are hardly usually detected by modern bioanalytical detection methods (Ewles and Goodwin, 2011). It is known that small molecular AMPs are rapidly metabolized *in vivo* and degraded into smaller peptide fragments or amino acids and absorbed as nutrients; thus, this situation becomes the blind zone for detection and characterization in trial research and licensing evaluation, although the safety of the degradation products of AMPs *in vivo* is unquestionable by general knowledge of nutrition metabolism but very difficult for demonstration *via* analytical detection. Surely, some new principles with more suitable for AMPs are urgent in need, specially for their clinical evaluation protocol. Usually, ELISA and LC–MS/MS analysis methods have been applied for quantifying typical peptides in biological samples (Ewles and Goodwin, 2011; Mercer and O'Neil, 2013), but not suitable for polar small AMPs. The microbiological assay (Chinese Veterinary Pharmacopeia, 2015, Appendix 1201), as a quantity- and function-based dual-label detection method, gives us a different solution facing above dilemma choice; it can concur and merge the determination of drug concentration and its antibacterial activity in biological samples, thus has been chosen for drug pharmacokinetic studies on AMPs, although it cannot clearly differentiate between a prototype drug and its active metabolites (Leroy et al., 1989; Jacobson et al., 2005; Andes et al., 2009; Hengzhuang et al., 2012; Giguère et al., 2017); in fact, it is well-suitable for characterizing the small molecular AMPs with a consensus among researcher peers. Gladly, similar supportive information can be seen from the regulation “Requirements for Application Materials of New Feed Additives of Announcement No. 226 of the Ministry of Agriculture and Rural Affairs of the P. R. China (MARA)”; if the compound or metabolic residue is a normal component of animal body fluid or tissue, or it is absorbed as nutrients according to the physiological mode and level of compounds *in vivo*, there is no need to evaluate its metabolism and residue in detail. This rule should also be invoked or extrapolated to the evaluation of the pharmacokinetics of AMPs. This would offer a wise way for most small molecular peptides, including AMPs during their access to legal official recognition *via* clinical evaluation. It is believed that the licensing authorities as CFDA, MARA, FDA, EMA, and PMDA would optimize the relative regulations to overcome the above difficulties, and the effective combination of the multiple pharmacokinetics analytical methods as ELISA, HPLC, LC–MS/MS, microbiological assay, and *in vivo* imaging might be expected.

Plectasin (MW 4398.80 Da) isolated from *Pseudoplectania nigrella* has potent antimicrobial activity against gram-positive bacteria *in vitro* and *in vivo*, especially for *Streptococcus pneumoniae*, which is superior to vancomycin and penicillin (Mygind et al., 2005; Schneider et al., 2010). However, plectasin had some limitations before clinical application, such as cytotoxicity and trypsin instability (Yang et al., 2019). NZX (MW 4383.89 Da), a variant of plectasin with three mutation sites (D9S, M13I, and K32R), had the increased net charge (from +1 to +2) and hydrophobicity (from −0.695 to −0.578) (Liu et al., 2020). Compared with rifampicin, NZX displayed more potent antibacterial activity against multi-drug resistant *Mycobacterium tuberculosis* (*M. tuberculosis*) (Tenland et al., 2018, 2019). NZX displayed a stronger activity against *Staptococcus hyicus* (*S. hyicus*) NCTC 10350 than ceftriaxone both *in vitro* and *in vivo* (Liu et al., 2020). In addition, NZX had low cytotoxicity, and good temperature, pH, and protease stability (Tenland et al., 2018; Liu et al., 2020). More importantly, NZX possessed the ability to induce intracellular killing of *M. tuberculosis* in human macrophages (Tenland et al., 2018) and *S. hyicus* NCTC 10350 in Hacat cells (Liu et al., 2020); thus, it provides a potential for the treatment of peritonitis diseases caused by intracellular bacterial infection.

In this study, the pharmacodynamics (PD) and pharmacokinetics (PK) of NZX against the *S. aureus*-induced mouse peritonitis model were built and evaluated for the first time; the main pharmacokinetic parameters and the relationships between PK/PD parameters and *in vivo* efficacies of NZX against total bacteria and intracellular bacteria were obtained, which will contribute to further clinical evaluation and application.

## Materials and Methods

### Chemicals, Organisms and Cell Line

NZX was expressed by *Pichia pastoris* X-33 and purified as described previously (Liu et al., 2020) (>90% purity). Antibacterial agents, including clindamycin, linezolid, daptomycin, and penicillin, were purchased from the China Institute of Veterinary Drug Control (Beijing, China), Dalian Meilun Biotech (Dalian, China), and Sangon Biotech (Shanghai, China) Co., Ltd. The clinical bovine mastitis-isolated *S. aureus* E48 was provided by Professor Xin Zhao, College of Animal Science and Technology, Northwest A&F University (Yangling, China). *S. aureus* MSSA ATCC 25923 and *S. aureus* MRSA ATCC 43300 were purchased from the American Type Culture Collection. The RAW 264.7 cell line was supplied by Peking Union Medical College.

### Minimal Inhibitory Concentrations (MICs) Against *S. aureus*

The MICs of NZX and other antibacterial agents against *S. aureus* strains were conducted according to the Clinical Laboratory Standards Institute (Watts et al., 2013). In brief, a volume of 90 μl of log-phase *S. aureus* E48 with a density of 1 × 10^5^ CFU/ml was co-incubated with 10 μl of NZX and antibiotics at final concentrations of 0.5–128 μg/ml and 0.125–64 μg/ml, respectively, including clindamycin, linezolid, daptomycin (with 1.25 mM of calcium chloride for daptomycin), and penicillin for 16–18 h at 37°C in a 96-well microplate. The wells containing antibiotics were set as positive controls. Negative control wells contained a PBS buffer and bacterial suspension without antibacterial agents. The MIC value was defined as the lowest concentration of antibacterial agents at which no visible bacteria were observed. All MIC assays were performed independently in triplicate.

### Bactericidal Activity and Drug Resistance Experiments

Survival rate and time-killing kinetic curves experiments were conducted as described previously (Li et al., 2020b). Log-phase *S. aureus* E48 cells with a density of 1 × 10^5^ CFU/ml were treated with NZX, clindamycin, linezolid, daptomycin (with 1.25 mM of calcium chloride for daptomycin), and ampicillin at 1/2 × MIC, 1 × MIC, and 2 × MIC, respectively. After incubation at various times (0, 15, 30, 60, 90, and 120 min), a volume of 100 μl of bacterial suspension was taken out from a flask and diluted to count. All assays were conducted independently in triplicate.

The development of drug resistance of *S. aureus* E48 induced by NZX and antibiotics was conducted as described previously (Li et al., 2017). The MICs of antibacterial agents were measured according to the MICs testing method as described above. After 16–18-h incubation at 37°C, the bacterial suspensions mixed with NZX, clindamycin, linezolid, daptomycin, and ampicillin at the sub-MIC were collected and transferred to 10 ml of an MHB medium until logarithmic growth. Then, the MIC values of the next passage were conducted again, and the experimental procedures were repeated for 30 days. The development of drug resistance was determined by the fold changes in MICs.

### Stability Under Different Physiological Environments

The stability of NZX in the mouse serum was determined when placed at 37°C (Wang et al., 2020). The stock mixture from 25% serum was spiked with 100 μg/ml NZX and vortexed well. The mixtures were incubated at 37°C. At 0, 4, 8, 12, and 24 h, samples were taken out, extracted, and analyzed by reverse-phase high-performance liquid chromatography (RP-HPLC). The results were expressed as the ratio of the peak area of the peptide at various times to that of the initial incubation time.

The physiological salt and serum stability of NZX were performed according to the previous report (Hirsch et al., 2019). In detail, the MIC values of NZX for *S. aureus* E48 were also determined in the MHB medium adjusted to 150-mM NaCl or 1.25-mM CaCl_2_, as well as in the MHB medium added into 25% (v/v) sterile mouse serum or in the MHB medium adjusted to 25% (v/v) mouse serum and supplemented with 150-mM NaCl, which represent the physiological salt and serum concentrations, respectively.

To test the effect of phagolysosomal enzyme-cathepsin B (from bovine spleen, Sigma C7800) on the antibacterial activities of peptides, NZX was diluted to 640 μg/ml in a cathepsin buffer (5-mM L-cysteine, 20-mM sodium acetate, 1-mM EDTA, pH of 5.0) and incubated with 16 μg/ml enzyme solution at 37°C for 1 h. The MIC value of NZX against *S. aureus* E48 was examined. NZX without enzyme solution was set as the negative control. Additionally, to mimic the intracellular condition, the MHB medium was adjusted to a specific pH value of 5.0 to determine the MIC value of NZX against *S. aureus* E48 in the acidic environment (Wang et al., 2019). In all experiments, clindamycin, linezolid, daptomycin, and ampicillin acted as antibiotic control.

### Cytotoxicity, Intracellular Inhibition, and Internalization Assays

The viability of RAW 264.7 cells was determined upon exposure to NZX by a colorimetric 3-(4, 5-dimethyl-2-thiazolyl)-2, 5-diphenyl-2-H-tetrazolium bromide (MTT) method as described previously (Li et al., 2021). All assays were repeated six times. The cell survival rate was calculated using the following formula:


$$\begin{array}{l} \text{Survival rate }\left(\%\right)\;=\frac{{\text{OD}}_{\text{compound}}-{\text{OD}}_{\text{blank}}\;}{{\text{OD}}_{\text{control}}-{\text{OD}}_{\text{blank}}}\times \;100\%\; \end{array}$$

The antibacterial effects of NZX against intracellular *S. aureus* E48 in RAW 264.7 were determined according to a previous study (Wang et al., 2018). The antibacterial concentrations of NZX against *S. aureus* E48 were set as 1, 4, 16, 64, and 128 μg/ml. Daptomycin was selected as a control because it is a polypeptide drug similar to NZX. In addition, the internalization and distribution assays of NZX in RAW 264.7 were conducted according to Wang et al. (2019). Briefly, RAW 264.7 cells were grown on culture dishes at 37°C for 24 h. FITC or FITC-NZX (1, 16, and 64 μg/ml) was added and incubated in the culture dishes for 24 h. The cells were washed two times with PBS and quenched by 0.04% trypan blue. Samples were analyzed by flow cytometry to determine the uptake of NZX. In distribution analysis, FITC or FITC-NZX at 64 μg/ml was added and incubated in the culture dishes for 24 h. The cells were washed two times with PBS, stained with 5 μg/ml wheat germ agglutinin (WGA)-conjugated Alexa Fluor 555 (ThermoFisher Scientific, USA) and Hoechst 33342 (ThermoFisher Scientific, USA) for 10 min, and observed by confocal microscopy (Leica TCS SP 5, Germany).

### Pharmacokinetics of NZX in Non-Infected Mice

The 6-week-old, specific-pathogen-free, female ICR mice (starting weight, 23 to 27 g) were purchased from Beijing Vital River Laboratory Animal Technology Co., Ltd (Beijing, China). The animals were acclimatized for 2 to 3 days before the experiment. The pharmacokinetics of NZX was evaluated in uninfected female ICR mice that received a single tail vein NZX dose of 5, 10, or 20 mg/kg, respectively. After NZX administration, blood samples were collected by orbital venous from five mice per time point at 0.083 to 2 h intervals over 6 h. After centrifugation at 4,000 rpm for 20 min at 4°C, the serum was immediately separated and frozen at −80°C until analysis. Analyses of total serum concentrations of NZX were conducted by a microbiologic assay using *S. aureus* ATCC 6538p (CICC 10307) as the test strain (Andes et al., 2009). The developed microbial method proved to be simple, precise, and stable. The limit of quantification (LOQ) and detection (LOD) in the microbiologic assay were 0.5 μg/ml and 0.25 μg/ml, respectively. The protein binding of NZX in serum was measured by the ultrafiltration method using Centrifree Filters (Merck Millipore Ltd., Product code: 4104), according to previously described methods by Brinch et al. (2009). The concentration of NZX in the ultrafiltrate was determined by HPLC methods.

### Pharmacodynamics of NZX in the Mouse Peritonitis Model

Each mouse was received a peritonitis by intraperitoneal injection of *S. aureus* E48 suspended in a PBS buffer (2 × 10^8^ CFU/ml, 0.5 ml). NZX treatment commenced 2 h after inoculation, with 5, 10, and 20 mg/kg doses given through tail vein one time or two times daily (2 h and 8 h after infection) (Liu et al., 2020). After therapy, the survival conditions of the mice were recorded within 7 days.

In this study, untreated mice that received a lethal dose of *S. aureus* (2 × 10^8^ CFU/ml, 0.5 ml) began to die at 6 to 8 h, and all died before 24 h, which made it difficult for us to obtain the samples when the therapy effects after 24 h administration were analyzed. Therefore, the mice that received half lethal doses of *S. aureus* E48 (1 × 10^8^ CFU/ml, 0.5 ml) were conducted in the following pharmacodynamics analysis. After 24 h therapy with NZX (5, 10, and 20 mg/kg) or daptomycin (20 mg/kg), organ tissues, including livers, lungs, spleens, hearts, and kidneys, were collected, homogenated, and plated to count bacterial burden. In addition, the total number of bacteria and intracellular bacteria counts in peritoneal fluids were determined as described by Wang et al. (2018). Briefly, the total bacterial CFU in the peritoneal fluid were quantified before any further procedures. In addition, the collected peritoneal fluid was centrifuged and washed to obtain peritoneal macrophage, and then extracellular bacteria were killed after treatment with 50 μg/ml lysostaphin at 37°C for 30 min. The cells were washed two times by PBS and suspended with 1 ml of cold sterile water for lysis, and the lysate was used to determine the intracellular bacterial burden.

### Pharmacokinetics and Pharmacodynamics Relationship

A dose fractionation study was applied to determine the PK/PD parameters of ƒAUC/MIC, ƒC_max_/MIC, and ƒT% > MIC, and those parameters were predictive of efficacies for NZX against *S. aureus* E48. Based on the PK study described above, NZX ranging from 5 to 40 mg/kg in 24 h was applied in order to change the PK/PD parameters of ƒAUC/MIC, ƒC_max_/MIC, and ƒT% > MIC (Table 1). The correlations between efficacies and each PK/PD index were analyzed by non-linear least-squares regression based on the sigmoidal E_max_ model (Hill's equation) (Yu et al., 2016). Hill's equation was defined as follows:


$$\begin{array}{l} \text{E }={\text{E}}_{\max}-\frac{\left({\text{E}}_{\max}-{\text{E}}_{0}\right)\;\times {{\text{ C}}_{\text{e}}}^{\text{N}}}{{{\text{EC}}_{50}^{\text{N}}\;+\;{\text{C}}_{\text{e}}}^{\text{N}}} \end{array}$$

where E is the observed antibacterial effect, which describes the change of log_10_ CFU/ml in the peritoneal fluid after 24 h of NZX treatment compared to the colony counts at the start of therapy. E_max_ is the change of log_10_ CFU/ml in the peritoneal fluid after 24 h of NZX treatment when the maximum antibacterial effect has been reached. E_0_ is the change of log_10_ CFU/ml in the peritoneal fluid between initial therapy and after 24 h therapy in untreated control animals. C_e_ is defined as the three PK/PD parameters (ƒAUC/MIC ratio, ƒC_max_/MIC ratio, and ƒT % > MIC). EC_50_ is the value of the PK/PD parameter that is required to achieve 50% of the maximum antibacterial effect. N is the steepness of the fitted concentration-response curve. The coefficient of determination (*R*^2^) between efficacy and the PK/PD parameters was used for determining the goodness of fit of the curves. In addition, the parameters required to achieve bacterial stasis (C_s_), 1 log_10_ kill, and 2 log_10_ kill were calculated interpolation or extrapolation based on Hill's equation.

**Table 1:** Treatment regimens and resulting PK/PD parameters for *S. aureus* E48 in the mouse peritonitis study.

**Total**	**Signal**	**No. of doses**	**fT > MIC (h)**	**fAUC/MIC**
**dose (mg)**	**dose (mg/kg)**	**in 24 h**		
0.5	20	1	2.05	8.89
1	20	2	4.09	17.79
0.25	10	1	0.94	3.54
0.5	10	2	1.88	7.08
0.125	5	1	0.33	1.11
0.25	5	2	0.67	2.22

### Statistical Analysis

The pharmacokinetic parameters of NZX in the serum were analyzed using a non-compartment model by WinNonlin software (version 8.1; Pharsight, USA). GraphPad Prism (version 9.0.1) was used for all statistical analyses. One-way ANOVA followed by Dunnett's test was performed to compare the difference between groups. A *P*-value <0.05 was considered as the significant difference. Kaplan Meier analysis was conducted for the survival curve; log-rank (Mantel-Cox) test analyses were used to compare the survival rate difference.

## Results

### Antibacterial Activity Against *S. aureus*

Antibacterial activity against clinical and reference *S. aureus* strains was shown in Table 2. NZX displayed stronger antibacterial activity against *S. aureus* MRSA ATCC 43300 (MIC = 1 μg/ml) than linezolid (MIC = 2 μg/ml) and ampicillin (MIC = 8 μg/ml), especially for clindamycin, which was inactive or only weakly active (MIC > 64 μg/ml), demonstrating that *S. aureus* MRSA ATCC 43300 is intrinsically resistant to clindamycin. Antibacterial activity of NZX (MIC = 1–2 μg/ml) against *S. aureus* MSSA ATCC 25923 and *S. aureus* E48 displayed that it was equivalent to linezolid (MIC = 1–2 μg/ml) and daptomycin (MIC = 0.5–2 μ*g*/ml), but inferior to clindamycin (MIC = 0.25 μg/ml) and ampicillin (MIC = 0.25 μg/ml).

**Table 2:** The MIC values of NZX and antibiotics against *S. aureus*.

**Drugs**	**MIC (μg/ml)**		
	**MRSA ATCC 43300**	**MSSA ATCC 25923**	**E48**
Clindamycin	>64	0.25	0.25
Linezolid	2	2	1
Daptomycin	0.5	2	1
Ampicillin	8	0.25	0.25
NZX	1	2	1

*The sequence of NZX: GFGCNGPWSEDDIQCHNHCKSIKGYKGGYCARGGFVCKCY (Liu et al., 2020)*.

*Reference strains: S. aureus MRSA ATCC 43300 and S. aureus MSSA ATCC 25923*.

*Clinical isolated strains: S. aureus E48*.

### Survival Rate and Development of Drug Resistance of *S. aureus* E48 *in vitro*

**Figure 1:**
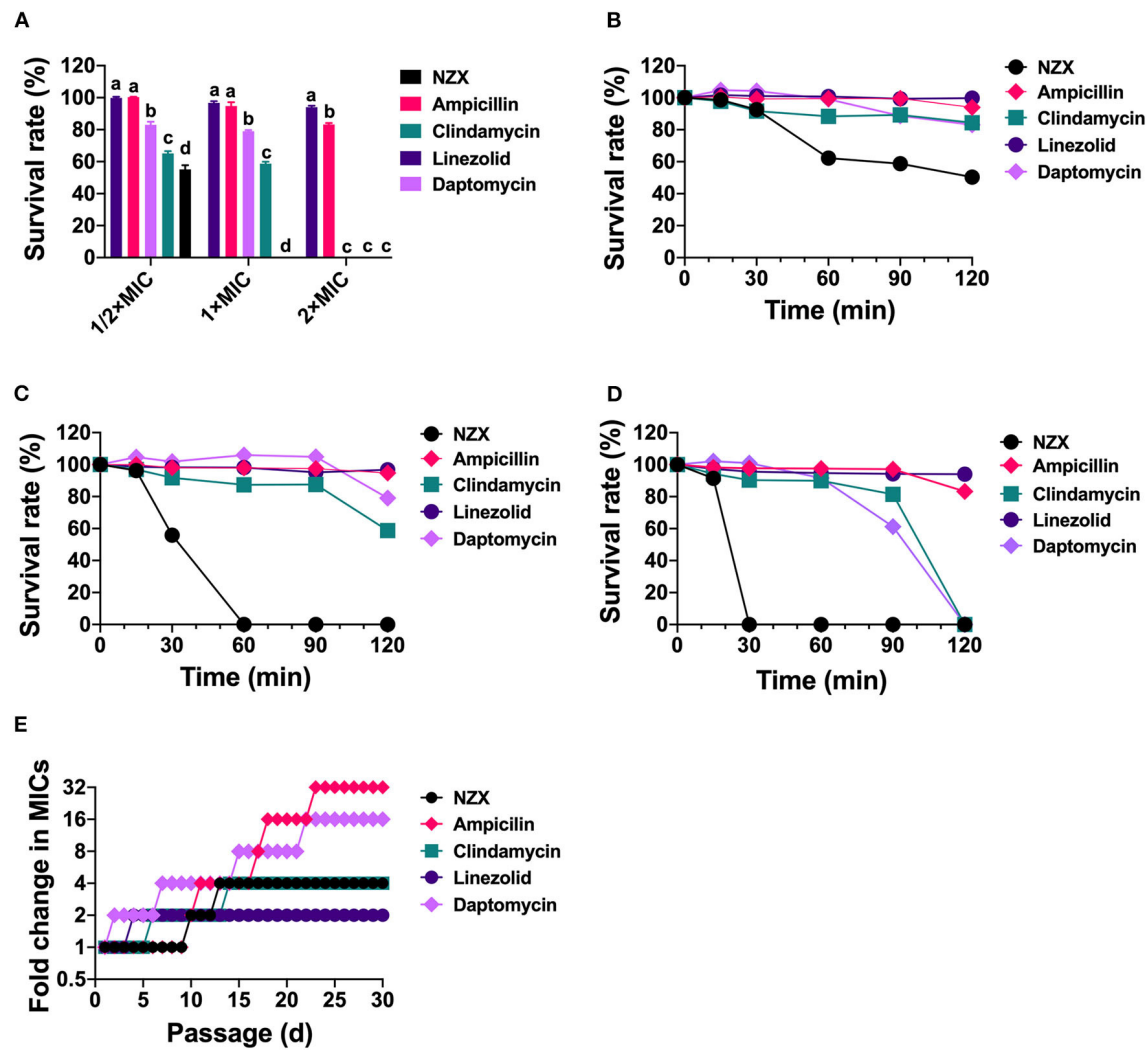
The survival rate and development of drug resistance of *S. aureus* E48 *in vitro*. **(A)** The bacterial cell survival ratio of *S. aureus* E48 treated with linezolid, ampicillin, daptomycin, clindamycin, and NZX at 1/2 × MIC, 1 × MIC, and 2 × MIC for 120 min. The statistical difference was conducted by one-way ANOVA, with Tukey's multiple comparisons test. Values are expressed as mean ± SD (*n* = 3), and different letters represent a significant difference in the same concentration (*p* <0.05). **(B–D)** The bacterial survival rate of *S. aureus* E48 treated with 1/2 × MIC **(B)**, 1 × MIC **(C)**, and 2 × MIC **(D)** linezolid, ampicillin, daptomycin, clindamycin, and NZX for 120 min. Values are expressed as means ± SD (*n* = 3). **(E)** Development of drug resistance of *S. aureus* E48 repeated exposure to linezolid, ampicillin, daptomycin, clindamycin, and NZX for 30-day with sub-MIC levels.

The antibacterial effects of NZX and antibiotics against *S. aureus* E48 compared to the initial incubation were shown in Figures 1A–D. Survival rates of *S. aureus* E48 treatment with linezolid, ampicillin, daptomycin, and clindamycin at 1/2 × −2 × MIC for 2 h were 94.08–99.89%, 83.21–100.31%, 0–83.03%, and 0–65.11%, respectively, which were inferior to those of NZX (0–50.35%) (Figure 1A). Specifically, after treatment with 1/2 × MIC NZX for 2 h or 1 × MIC NZX for 1 h, *S. aureus* was killed by 49.65% (Figure 1B) or 100% (Figure 1C), respectively, which all showed more potent and rapid bactericidal activity than those four chosen antibiotics. In addition, after incubation with 2 × MIC NZX for 0.5 h, *S. aureus* was already completely killed; however, the clindamycin and daptomycin groups eliminated bacteria within 2 h, which displayed more efficient activity than linezolid and ampicillin (Figure 1D).

Development of drug resistance of *S. aureus* E48 was shown in Figure 1E. Results showed that MIC values of linezolid, NZX, clindamycin, daptomycin, and ampicillin against *S. aureus* E48 after 30-day serial passage were 2, 4, 1, 8, and 16 μg/ml, which resulted in a 2-, 4-, 4-, 16-, and 32-fold increase in the MIC value, respectively. Those results indicated that NZX had a lower risk of resistance compared with ampicillin and daptomycin.

### Stability of NZX Under Different Physiological Conditions

**Figure 2:**
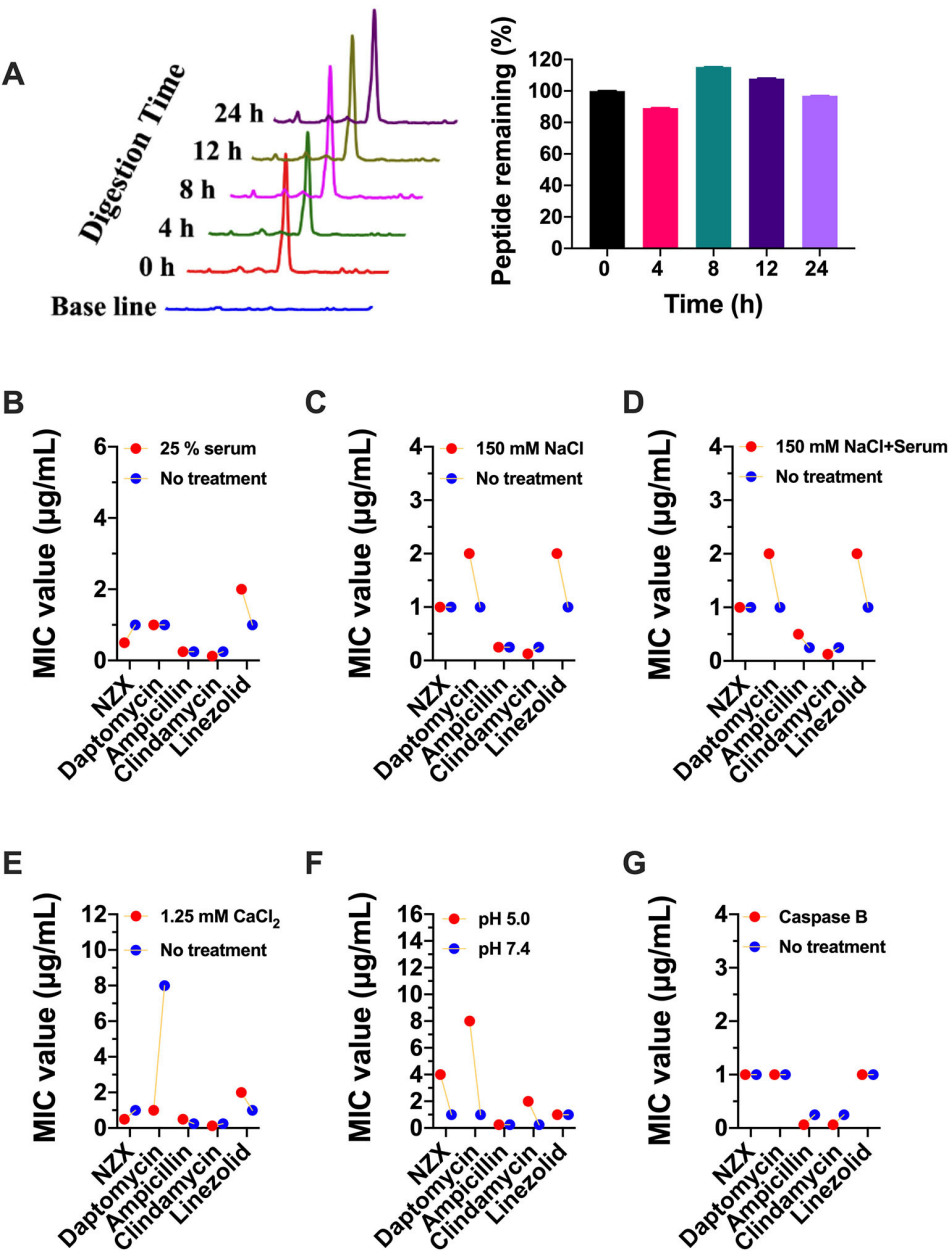
Stability of NZX under different physiological conditions. **(A)** The peptide remaining after incubation with 25% serum for 24 h in serum. (Left) chromatogram; (Right) histogram. **(B–G)** The activity of NZX exposure to different physiological conditions for 24 h. No treatment: with the only presence of the MH medium.

The peptide remaining showed that 96.99% of NZX still remained intact after incubation with 25% serum for 24 h (Figure 2A). In addition, there was no change or just 2-fold in the MIC values of NZX and four antibiotics under 25% serum (Figure 2B). Similarly, the MIC values of NZX and four antibiotics in physiological salt environments were unchanged or just 2-fold change, except for daptomycin, which displayed 8–16-fold decrease under the addition of 1.25-mM CaCl_2_ (Figures 2C–E). The MIC values of NZX, daptomycin, and clindamycin in intracellular lysosomal acid environment at pH 5.0 were up to 4-, 8-, and 8-fold when compared to those of pH 7.4; however, the antibacterial activities of ampicillin and linezolid did not influence (Figure 2F). Furthermore, the antibacterial activities of NZX, daptomycin and linezolid did not change in phagolysosomal proteinase environments, but the MIC values of ampicillin and clindamycin were all down to at least 4-fold (Figure 2G).

### Low Toxicity, Intracellular Bactericidal Activity, Uptake, and Location of NZX

**Figure 3:**
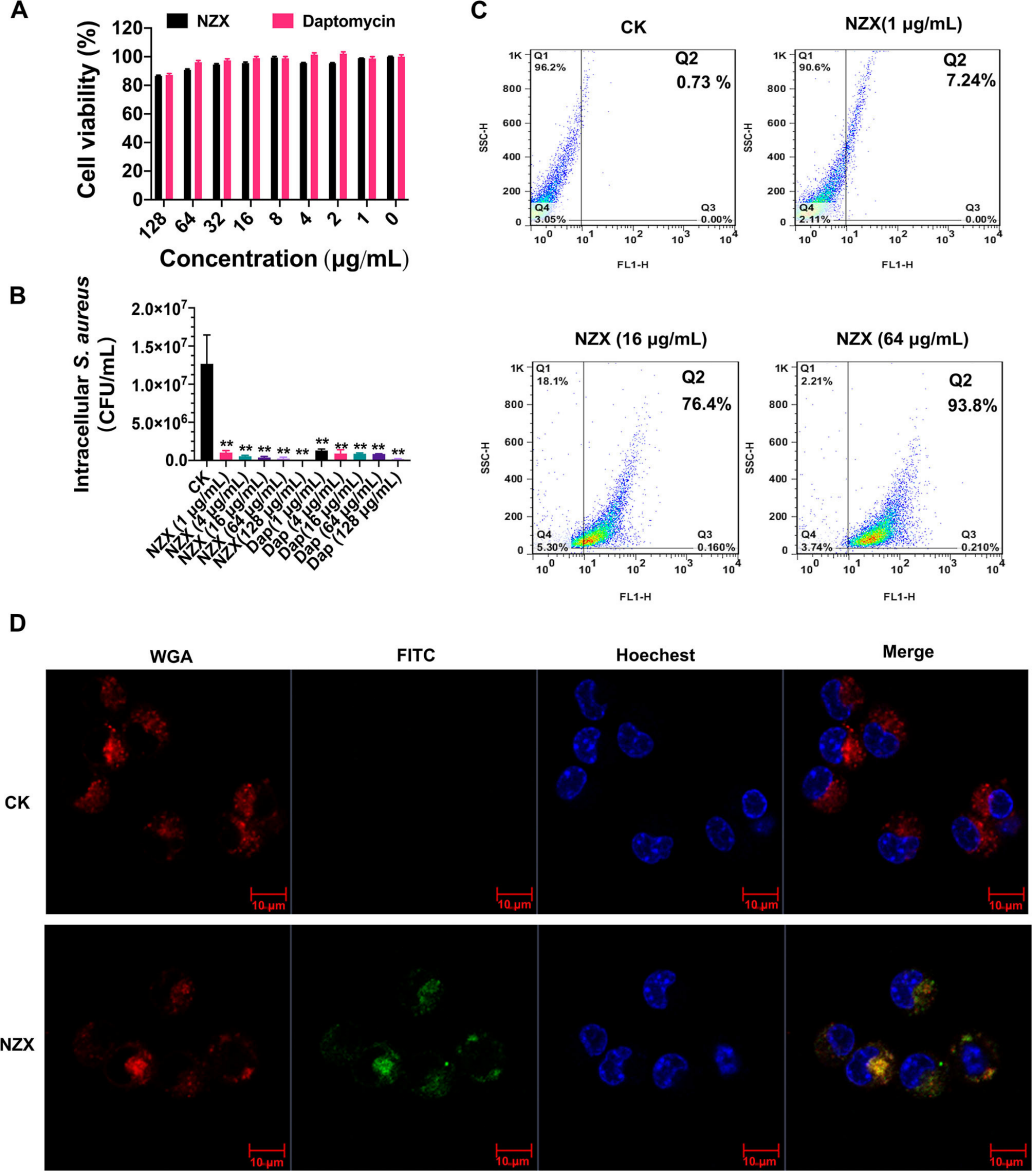
Cell toxicity, intracellular antibacterial activity, cellular uptake, and distribution of NZX in RAW 264.7 cells. **(A)** Cell viability of RAW 264.7 cells incubated with NZX with concentrations ranging from 1 to 128 μg/ml for 24 h by the MTT method. Daptomycin was set as a positive control. Results from three experiments were expressed as means ± SD (*n* = 3). **(B)** Intracellular antibacterial activity of NZX in RAW 264.7 cells infected with *S. aureus* E48. The activity of NZX and daptomycin against intracellular *S. aureus* E48 in RAW 264.7 cells was measured after treatment for 24 h. CK: without the presence of any antibacterial agents. **(C)** Cellular uptake of FITC-labeled NZX. The cells were incubated with 1-, 16-, and 64 μg/ml FITC-labeled NZX for 24 h. The internalization ratio of intracellular FITC-labeled NZX was determined by setting quadrants on the flow cytometry. **(D)** FITC-labeled NZX distribution in RAW 264.7 cells. The cells were incubated with 64 μg/ml FITC-labeled NZX for 24 h. The localization of FITC-NZX was visualized by confocal microscopy. CK: free FITC without conjugated to NZX. The cells incubated with FITC-NZX presented a green signal; the cell nucleus and membrane were stained with Hoechst 33342 (a blue signal) and wheat germ agglutinin (WGA)-Alexa Fluor® 555 (a red signal), respectively.

As shown in Figure 3A, the viability of RAW 264.7 cells was 85.56~98.96% after treatment with 1~128 μg/ml NZX for 24 h. Furthermore, after exposure to 1~128 μg/ml daptomycin, the cell viability was 87.22~102.19%, which was similar to the NZX treatment. These results demonstrated that NZX and daptomycin had low toxicity to RAW 264.7 cells at tested concentrations.

The intracellular anti- *S. aureus* capacity of NZX and daptomycin were determined at different concentrations (1, 4, 16, 64, and 128 μg/ml) using RAW 264.7 cells. As shown in Figure 3B, NZX was effective in mediating intracellular *S. aureus* load reduction in a concentration-dependent manner with reductions of 91.81~99.81% of intracellular bacteria. Furthermore, at the same concentrations (1, 4, 16, 64, and 128 μg/ml), daptomycin eliminated approximately 89.74~98.53% of intracellular *S. aureus*. The intracellular bacteria treated with NZX and daptomycin over a wide range of concentrations were all significantly reduced compared with the CK group (Figure 3B). In addition, higher killing potency of NZX against intracellular *S. aureus* at 16, 64, and 128 μg/ml concentrations was determined compared to the same concentration of daptomycin treatment (Figure 3B).

The uptake efficiency of NZX into RAW 264.7 cells was determined by flow cytometry. After treatment with 1, 16, and 128 μg/ml FITC-NZX for 24 h, the FITC-labeled NZX entered the cell in a dose-dependent way, reaching up to 7.24~93.8% (Figure 3C). Confocal microscopy analysis was further performed to determine the subcellular distribution of NZX in RAW 264.7 cells. As shown in Figure 3D, FITC-labeled NZX was distributed in cell cytosol in a cluster manner, suggesting that the uptake of NZX might be *via* macropinocytosis and/or endocytosis.

### Pharmacokinetics of NZX

**Figure 4:**
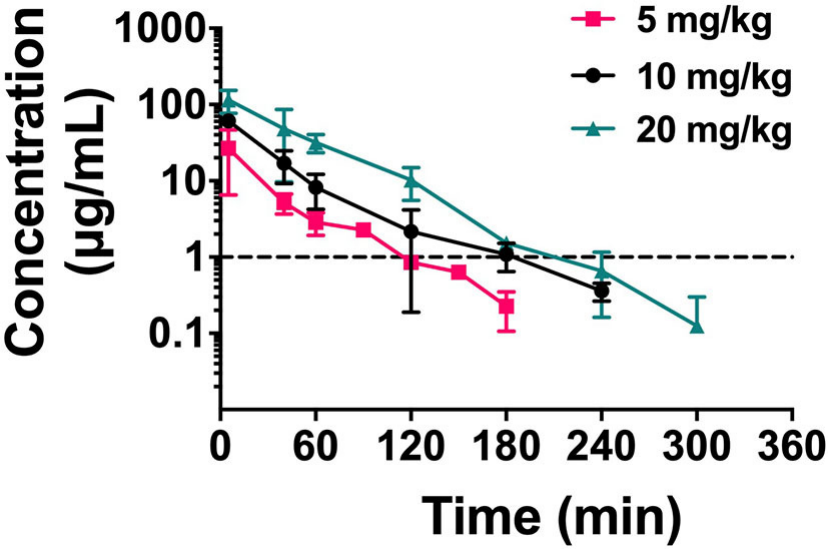
The drug concentration-time curve of NZX. Free serum concentrations for NZX after single intravenous administration of NZX at escalating doses of 5-, 10-, and 20 mg/kg body weight in non-infected mice. Each symbol from five mice indicates the Mean ± SD. The dotted line represents the MIC value of NZX against *S. aureus* E48.

Single-dose PK studies of NZX were conducted in non-infected mice. The free drug serum concentrations-time curves of NZX for 6 h following intravenous doses of 5, 10, and 20 mg/kg was shown in Figure 4, and the corresponding PK parameters for NZX were listed in Table 3. Peak concentrations (C_max_) were observed at 5 min after intravenous administration. The area under concentration-time curves (AUC) and C_max_ values for the three-dose levels ranged from 1.11 to 8.89 μg·h/ml and 2.85 to 20.55 μg/ml, which were linear over the dose range with a coefficient of determination of 0.9995 and 0.9818, respectively. The elimination half-lives (T_1/2_) over the three different dose levels ranged from 0.32 to 0.45 h. In addition, the PK parameters for apparent distribution volume (V_d_) and mean residence time (MRT) ranged from 1469.10 to 2073.90 ml/kg and 0.32 to 0.56 h, respectively. The murine serum protein binding of NZX was measured to be 89.25% by ultrafiltration method.

**Table 3:** Parameter estimates from PK analysis of free-serum NZX concentrations after single intravenous administration of an NZX dose of 5 to 20 mg/kg.

**Parameter**	**Units**	**Estimates**		

**Dose**	**mg/kg**	**5.00**	**10.00**	**20.00**
T_1/2_	h	0.32	0.50	0.45
C_max_	μg/ml	2.85	6.53	20.55
V_d_	mL/kg	2073.90	2045.41	1469.10
MRT	h	0.32	0.64	0.56
AUC	μg.h/ml	1.11	3.54	8.89
Protein binding	%	89.25		

*T_1/2_, terminal elimination half-life; C_max_ peak concentration; V_d_, apparent distribution volume; MRT, mean residence time; AUC, area under concentration-time curves*.

### Pharmacodynamics of NZX Against the *S. aureus*-Induced Mouse Peritonitis Model

**Figure 5:**
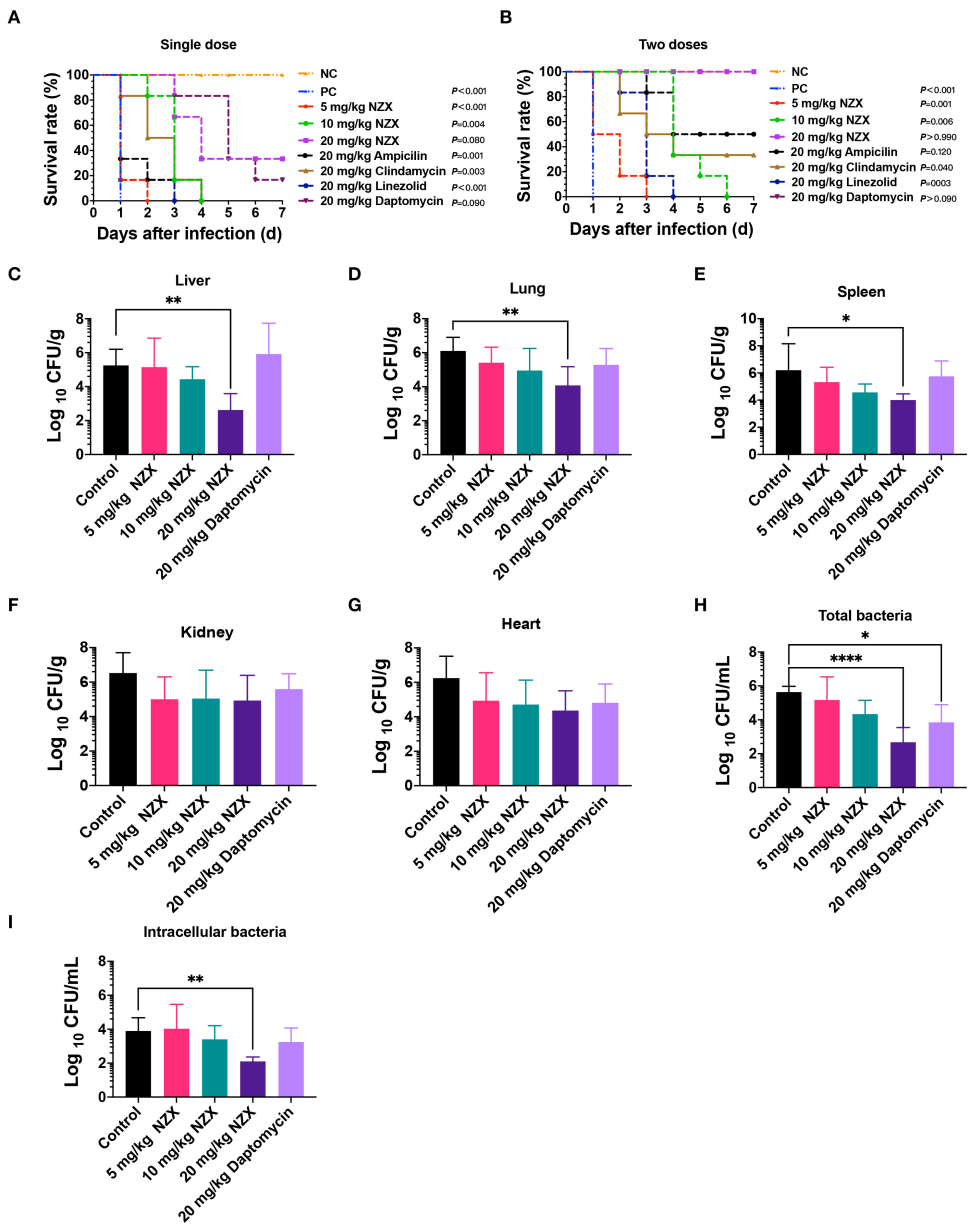
Pharmacodynamics of NZX against *S. aureus*-induced mouse peritonitis model. **(A,B)** The survival rate of ICR mice (*n* = 6) that were challenged intraperitoneally with *S. aureus* E48 (2 × 10^8^ CFU/ml, 500 μl) for 7-day post-infection. NZX at doses of 5, 10, and 20 mg/kg were administered intravenously single dose **(A)** (2 h) and two doses **(B)** (2 and 8 h) post-infection. The PC group: the infected but untreated mice; NC: the uninfected mice. **(C–G)** The bacterial burden of organ tissues and peritoneal fluid in the ICR mice infected intraperitoneally with *S. aureus* E48 (1 × 10^8^CFU/ml, 500 μl). The bacterial counts in the liver **(C)**, lung **(D)**, spleen **(E)**, kidneys **(F)**, heart **(G)**, total bacteria **(H)**, and intracellular bacteria **(I)** in the peritoneal fluid were analyzed after treatment with NZX (5, 10, and 20 mg/kg) or daptomycin (20 mg/kg) for 24 h. The untreated mice were used as the negative control. The statistical difference was conducted by one-way ANOVA, with Tukey's multiple comparisons test. Values are expressed as means ± SD (*n* = 6); *P*-value <0.05 was considered significant; *p <0.05; **p <0.01; ****p <0.0001.

In the mouse peritonitis model, ICR mice were intraperitoneally infected with *S. aureus* E48 to evaluate the therapeutic efficacy of NZX. As shown in Figures 5A,B, the untreated mice were all dead within 24 h after infection. Although after treatment with NZX (5 and 10 mg/kg) and antibiotics (20 mg/kg), including linezolid, ampicillin, and clindamycin by intravenously single-time injection, all the mice were dead; the survival time of the mice extended for 2–4 days (Figure 5A). After treatment with 20 mg/kg NZX, the survival rate of the mice was 33.3%, which was higher than that of treatment with 20 mg/kg daptomycin (16.7%) (Figure 5A). Interestingly, after two-times intravenous injection with NZX and daptomycin at a dose of 20 mg/kg (2 h and 8 h after infection, respectively), both groups of the mice all survived (100%), but the survival rates of the mice treated with ampicillin and clindamycin were 50% and 33.3%, respectively (Figure 5B). In addition, treatment with 5 and 10 mg/kg NZX and 20 mg/kg linezolid did not improve the survival rate of the mice, but all extended lives by 2–6 days (Figure 5B).

The bacterial implantation levels in the liver, lung, and spleen after 24 h were 5.25 ± 0.96, 6.10 ± 0.80, and 6.21 ± 1.95 Log_10_ CFU/g, respectively (Figures 5C–E). After treatment with NZX, CFU counts in liver, lung, and spleen reduced by −0.10 ± 1.70~2.63 ± 0.97 Log_10_CFU/g, 0.69 ± 0.91~2.03 ± 1.10 Log_10_ CFU/g, and 0.88 ± 1.09~2.20 ± 0.46 Log_10_ CFU/g, respectively, which displayed a dose-dependent effect (Figures 5C–E). Comparing NZX at the 20 mg/kg treatment group with the untreated group, all significant decrease in bacteria burdens of those tissues was observed (Figures 5C–E). Nevertheless, no obvious difference between daptomycin and the untreated group was observed (Figures 5C–E). In addition, therapeutic effects of NZX against organ tissues of the kidneys and heart were also studied, but bacteria burdens of those tissues did not get obviously decreased (Figures 5F,G).

The therapeutic effects of NZX or daptomycin on the total bacteria and intracellular bacteria in the peritoneal fluid were shown in Figures 5H,I. Total bacterial and intracellular bacteria burdens of the untreated mice in the peritoneal fluid were 5.64 ± 0.34 and 3.90 ± 0.78 Log_10_ CFU/ml (Figures 5H,I). After treatment with NZX at doses of 5, 10, and 20 mg/kg, the total bacterial and intracellular bacteria burdens reduced by 0.47 ± 1.37~2.96 ± 0.87 Log_10_ CFU/ml and−0.13 ± 1.44~1.79 ± 0.26 Log_10_ CFU/ml, respectively, and the 20 mg/kg NZX treatment group displayed significant difference with the untreated group, which showed a concentration-dependent effect (Figures 5H,I). The total bacterial and intracellular bacteria burdens of 20 mg/kg daptomycin reduced by 1.79 ± 1.05 Log_10_ CFU/ml and 0.66 ± 0.83 Log_10_ CFU/ml, respectively (Figures 5H,I). A significant decrease between daptomycin and the untreated group was observed in the total bacteria, but no significant difference in intracellular bacteria (Figures 5H,I).

### Pharmacokinetics and Pharmacodynamics Index Determination

**Figure 6:**
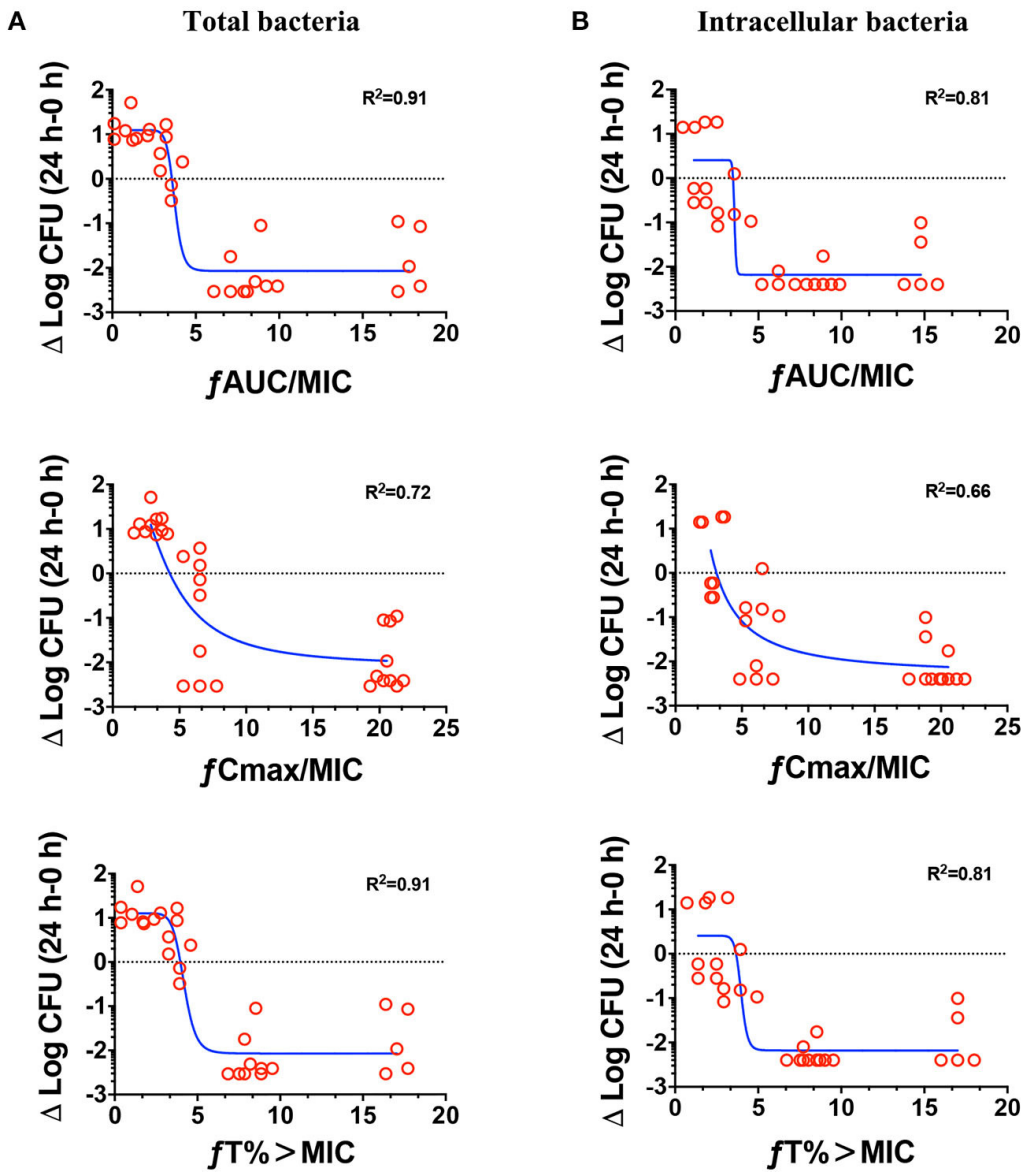
Pharmacokinetics and pharmacodynamics index determination. Correlations between the pharmacokinetic parameters (24 h ƒAUC/MIC, ƒC_max_/MIC, and ƒT% > MIC) and efficacies for total bacteria **(A)** and intracellular activity **(B)** over 24 h against *S. aureus* E48 (MIC = 1 μg/ml) in the mouse peritonitis model. The ordinate indicated the total bacteria and intracellular bacteria counts change in peritoneal lavage fluids between the treated for 24 h animals and those at the start of therapy. Each symbol indicates the bacteria of each mouse. The curves indicate the best fit using the sigmoid E_max_ model. *R*^2^ value represents the magnitude of correlation.

The relationships between the PK/PD parameters and efficacy were presented in Figure 6, and the corresponding pharmacological descriptors were shown in Tables 4, 5, respectively. Maximal effects (E_max_) were observed for NZX against total bacteria and intracellular bacteria, which correspond to or close to the actual limit of detection (Figure 6 and Table 5). Results showed that the strongest relationship between the three PK/PD parameters (ƒAUC/MIC, ƒT% > MIC, and ƒC_max_ > MIC) and NZX efficacy against total bacteria was observed, with *R*^2^ values of 0.91, 0.91, and 0.72, respectively (Table 4). Similarly, the strongest relationship between the three PK parameters and efficacy against intracellular bacteria was noted, with *R*^2^ values of 0.81, 0.81, and 0.66, respectively (Table 4). The parameters required to achieve bacterial stasis (C_s_), 1 log_10_ kill and 2 log_10_ kill were shown in Table 5. The static dose ƒAUC/MIC ratio, the ƒT% > MIC ratio, and the ƒC_max_ > MIC ratio for total bacteria were 3.574, 4.274, and 3.971, respectively (Table 4). The 1 log_10_ kill ƒAUC/MIC ratio, the ƒT% > MIC ratio, and the ƒC_max_ > MIC ratio for total bacteria were 3.896, 4.434, and 6.595, respectively (Table 5). The 2 log_10_ kill ƒAUC/MIC ratio, the ƒT% > MIC ratio, and the ƒC_max_ > MIC ratio for total bacteria were 4.778, 5.759, and 24.492, respectively (Table 5). Moving to intracellular bacteria, the static dose ƒAUC/MIC ratio, the ƒT% > MIC ratio, and the ƒC_max_ > MIC ratio were 3.473, 3.670, and 3.090, respectively (Table 5). The 1 log_10_ kill ƒAUC/MIC ratio, the ƒT% > MIC ratio, and the ƒC_max_ > MIC ratio were 3.569, 4.010, and 4.734, respectively (Table 5). The 2 log_10_ kill ƒAUC/MIC ratio, the ƒT% > MIC ratio, and the ƒC_max_ > MIC ratio were 3.698, 4.502, and 13.877, respectively (Table 5).

**Table 4:** Parameters for mean Hill-curve fit for total bacteria and intracellular bacteria.

**Parameters**	**Total bacteria**			**Intracellular bacteria**		
	**EC_**50**_**	**E_**max**_**	***R* ^2^**	**EC_**50**_**	**E_**max**_**	***R* ^2^**
ƒAUC/MIC	3.727	−2.071	0.91	3.560	−2.179	0.81
ƒC_max_/MIC	3.635	−2.077	0.72	0.261	−2.303	0.66
ƒT% > MIC	4.188	−2.071	0.91	3.976	−2.179	0.81

**Table 5:** The parameters required to achieve bacterial stasis or 1 log_10_ kill or 2 log_10_ kill for total bacteria and intracellular bacteria.

**Parameters**	**Total bacteria**			**Intracellular bacteria**		
	**Stasis**	**1 log_**10**_ kill**	**2 log_**10**_ kill**	**Stasis**	**1 log_**10**_ kill**	**2 log_**10**_ kill**
ƒAUC/MIC	3.574	3.896	4.778	3.473	3.569	3.698
ƒC_max_/MIC	4.274	6.595	24.492	3.090	4.734	13.877
ƒT% > MIC	3.971	4.434	5.795	3.976	4.010	4.502

*Stasis: The parameters resulting in no apparent bacterial growth in total bacteria and intracellular bacteria after 24 h treatment compared to the initial incubation*.

*1 log_10_ kill: The parameters resulting in decrease of 1 log_10_ CFU/ml in total bacteria and intracellular bacteria after 24 h treatment compared to the initial incubation*.

*2 log_10_ kill: The parameters resulting in decrease of 2 log_10_ CFU/ml in total bacteria and intracellular bacteria after 24 h treatment compared to the initial incubation*.

## Discussion

NZX, a variant of plectasin, has garnered interest as an ideal therapeutic drug against *S. aureus* because of its ability to kill intracellular bacteria and good biocompatibility (Tenland et al., 2018; Liu et al., 2020). Compared with plectasin (MIC, 4 μg/ml), NZX displayed a 4-fold higher antimicrobial activity (Table 2) (Yang et al., 2019). Furthermore, plectasin at a concentration of 2 × MIC killed approximately 85.85% of *S. aureus* E48 within 1 h, and eliminated bacteria within 2 h, which exhibited inferior bactericidal activity compared with NZX (Figures 1A–D) (Yang et al., 2019). This is because that replacement of D, M, and K residues in plectasin at positions 9, 13, and 31 by S, I, and R increased the net charge and hydrophobicity of NZX (Liu et al., 2020). Increased net charge and hydrophobicity improved the antibacterial activity of peptides (Higgs et al., 2007; Zhou et al., 2019; Tan et al., 2021); thus NZX showed more effective and rapid antibacterial activity than plectasin at the same concentration.

Unlike conventional antibiotics, AMPs usually kill pathogenic bacteria by physically disrupting the cell membrane, causing leakage of contents, and interfering with intracellular metabolic pathways (Gan et al., 2021; Jiang et al., 2021). Due to the unique mechanism of action, AMPs are not easy to cause bacterial mutations or develop only limited resistance with fitness cost (Choi and Ko, 2015; Nang et al., 2018; Gan et al., 2021; Jiang et al., 2021). Spohn et al. (2019) systematically investigated the resistance evolution of 14 AMPs and 12 antibiotics against *Escherichia coli in vitro*, demonstrating that AMPs had a lower risk of resistance than those of antibiotics (Spohn et al., 2019). Similarly, in this study, NZX had a lower risk of resistance compared with those of ampicillin and daptomycin (Figure 1E). Drug stability is vital for clinical application. NZX displayed good tolerance to serum, physiological salt, and proteinase conditions, but poor in the phagolysosomal acid environment (Figure 2), which were in accordance with the previous studies (Wang et al., 2018; Li et al., 2021).

The intracellular survival mechanism of *S. aureus* contributes to pathogen dissemination among organ tissues, resulting in disease progression or recurrence (Löffler et al., 2014; Horn et al., 2018; Wang et al., 2021) and even being at a chronic situation. Plectasin, NZ2114, and MP1102 can kill intracellular *S. aureus* in human THP-1 macrophages and RAW 264.7 cells, respectively (Brinch et al., 2009, 2010; Wang et al., 2018). NZX also significantly reduced the intracellular *S. aureus* in RAW 264.7 cells (Figure 3B). Different from intracellular activities of NZ2114 (Brinch et al., 2010), NZX exhibited higher killing potency than that of daptomycin. Entering into host cells and contacting with the pathogen are prerequisites of drugs for intracellular *S. aureus* elimination (Carryn et al., 2003). Both MP1102 and NZ2114 can enter into RAW 264.7 cells in a cluster manner and a dose-dependent way, and distributed in the cytoplasm (Wang et al., 2018). Similarly, NZX was distributed and internalized in the same manner (Figures 3C,D). In addition, internalization mechanisms exhibited that the entrance of MP1102 and NZ2114 into RAW 264.7 cells were mainly dependent on clathrin-mediated endocytosis and micropinocytosis in an energy- and temperature-dependent manner (Wang et al., 2018). As an analog of NZ2114 and MP1102 peptide, so should be NZX, but further research is needed.

In this study, the protein-binding degree of NZX (89.25%) was similar to plectasin (90%) in human and mice serum, and higher than that of NZ2114 (80%) (Andes et al., 2009; Brinch et al., 2009), indicating that it could bind to serum protein components, such as albumin, apolipoproteins or glycoproteins. As the dominant role of those free constituents in pharmacodynamics and pharmacokinetics, a plethora of studies indicated that the binding degree of drugs to serum protein will influence their pharmacological parameters and antibacterial activity, but those impacts on their activity were multifaceted and more complicated (Zeitlinger et al., 2008; Beer et al., 2009; Dalhoff, 2018). Some studies demonstrated that antibacterial agents with high protein binding had a potent and enhanced bactericidal ability, and some drugs above 90% plasma protein binding had also been approved, which did not disturb its suitability for systemic application (Perl et al., 1990; Cheah et al., 2015; Dalhoff, 2018; Hirsch et al., 2019). Similar to plectasin and NZ2114 with short half-lives (Andes et al., 2009; Brinch et al., 2009), NZX was rapidly metabolized by a mouse (Figure 4). Owing to peptides typically with <10 kDa in molecular mass, they could be freely filtered by the kidney and glomerulus, and were easily metabolized or degraded by the ubiquitous proteases (Lin, 2009; Meibohm and Zhou, 2012; Diao and Meibohm, 2013). These characteristics might answer the questions from the antibacterial peptide in preclinical pharmacokinetics. To overcome quick elimination of NZX in mouse serum, some strategies extending half-life have been tried to improve the pharmacokinetic characteristics of peptides (Diao and Meibohm, 2013; Kanugo and Misra, 2020).

The ability of NZX to decrease bacterial burden in organ tissues depends on its distribution. Benincasa et al. demonstrated that Bac7(1-35)-Alexa680 peptide *via* intraperitoneal injection could only reach kidneys and liver (Benincasa et al., 2010). Bailleul et al. demonstrated fluorescence-labeled AvBD7 by intraperitoneal injection could reach kidneys, liver, spleen, and mesenteric lymph nodes (Bailleul et al., 2019). Different from those reports (Benincasa et al., 2010; Bailleul et al., 2019), a substantial amount of NZX was observed in the kidneys and spleen at a rank of the second and third most fluorescent organs after the lung by *in vivo* imaging (Supplementary Figure S1), which were in accordance with the antibacterial activity of NZX in those organs (Figures 5C–G). That means that each AMP distribution in body depends on its individual characteristics. We know that intracellular drug concentration is vital to the treatment of intracellular bacteria, but it is extremely difficult to obtain intracellular pharmacokinetic characteristics of drugs *in vivo* (Levison and Levison, 2009). Therefore, serum drug levels were usually used as the first choice for predicting intracellular concentrations regardless of their correlation (Levison and Levison, 2009). Different from Brinch et al. (2009), who demonstrated that ƒC_max_/MIC was the most important parameter for the activity of plectasin against total bacteria and intracellular bacteria, we found that all three PK/PD parameters showed the strongest correlation with efficacies (Figure 6 and Table 4). In general, our work mainly focused on the relationship between efficacy and PK/PD parameters against *S. aureus* in a mouse peritonitis model; their direct comparisons to other peptides need more data from different animal models in the future.

## Conclusion

NZX displayed a narrow antimicrobial spectrum, high potency, and rapid antibacterial activity against the tested *S. aureus* with a lower frequency of resistant mutations compared with ampicillin and daptomycin. Stability results *in vitro* showed that NZX displayed good biocompatibility and tolerance to physiological salt, serum, and phagolysosomal cathepsin, except for the phagolysosomal acid environment. In addition, NZX could enter into RAW 264.7 cells in a dose-dependent manner and exhibited higher killing potency against intracellular *S. aureus in vitro* than daptomycin. Pharmacokinetics results of NZX *in vivo* showed that serum protein binding was 89.25%, and corresponding parameters of the AUC and C_max_ and T_1/2_ for the escalating NZX doses ranged from 1.11 to 8.89 μg.h/ml, 2.85 to 20.55 μg/ml, and 0.32 to 0.5 h, respectively. Together with the pharmacodynamics results *in vivo*, NZX administered intravenously two times could rescue all infected mice equivalent to daptomycin and NZX treatment, significantly reduced CFU counts in organ tissues and in the peritoneal fluid, which were superior or similar to those of treatment with daptomycin. These *in vivo* efficacies were significantly correlated with three PK/PD indices of ƒAUC/MIC, ƒC_max_/MIC, and ƒT% > MIC, as analyzed by a sigmoid maximum-effect model. Altogether, this study demonstrated that NZX could act as a new antimicrobial agent to protect host against infectious diseases caused by *S. aureus*.

## Data Availability Statement

The original contributions presented in the study are included in the article/Supplementary Material, further inquiries can be directed to the corresponding author/s.

## Ethics Statement

The animal study was reviewed and approved by the Animal Care and Use Committee of the Feed Research Institute, Chinese Academy of Agricultural Sciences (Permit Number: 20150309).

## Author Contributions

XZ, DT, and JW conceived and designed the experiments. XZ carried out all the experiments. XZ, DT, and NY guided the methods and contributed to writing. YH and RM contributed materials and reagents. JW contributed to funding acquisition. All the authors contributed to the article and approved the submitted version.

## Funding

This work was supported by the National Natural Science Foundation of China (Grant No. 31872393), the Innovation Program of Agricultural Science and Technology AMPs and Alternatives to Antibiotics for Animal Usage (ASTIP) in CAAS (Grant No. CAAS-ASTIP-2013-FRI-02), and its key projects (Grant Nos. CAAS-ZDXT201808 and CAAS-ZDRW202111).

## Conflict of Interest

The authors declare that the research was conducted in the absence of any commercial or financial relationships that could be construed as a potential conflict of interest.

## Publisher's Note

All claims expressed in this article are solely those of the authors and do not necessarily represent those of their affiliated organizations, or those of the publisher, the editors and the reviewers. Any product that may be evaluated in this article, or claim that may be made by its manufacturer, is not guaranteed or endorsed by the publisher.
