# Supplementary material for: Pharmacokinetics and Pharmacodynamics of Fungal Defensin NZX Against Staphylococcus aureus-Induced Mouse Peritonitis Model
Source: Front Microbiol. 2022 Jun 1;13:865774. doi: 10.3389/fmicb.2022.865774 (PMC9198545; doi:10.3389/fmicb.2022.865774)
Supplement: Supplementary file 1 [file Data_Sheet_1.pdf]

Frontiers in Microbiology

Supplementary Material

Pharmacokinetics and Pharmacodynamics of Fungal Defensin NZX against *Staphylococcus aureus*-induced Mouse Peritonitis Model

Xueling Zheng[1,2,3], Na Yang[1,2,3], Ruoyu Mao[1,2,3], Ya Hao[1,2,3], Da Teng[1,2,3†] and Jianhua Wang[1,2,3†]

[1] Gene Engineering Laboratory, Feed Research Institute, Chinese Academy of Agricultural Sciences, Beijing 100081, China

[2] Innovative Team of Antimicrobial Peptides and Alternatives to Antibiotics, Feed Research Institute, Chinese Academy of Agricultural Sciences, Beijing 100081, P. R. China

[3] Key Laboratory of Feed Biotechnology, Ministry of Agriculture and Rural Affairs, Beijing 100081, China

[†] Corresponding authors

(1) Prof., Ph.D., and PI. Jianhua Wang, and postal address of all authors as:
Gene Engineering Laboratory, Feed Research Institute
Chinese Academy of Agricultural Sciences, 12 Zhongguancun Nandajie St., Haidian District, Beijing 100081, P. R. China
E-mail address: wangjianhua@caas.cn; wangjianhua.peking@qq.com
Phone: 0086-10-82106081, 0086-10-82106079; Fax: 0086-10-82106079
(2) Prof. and Ph.D. Da Teng, e-mail address: tengda@caas.cn

Materials and Methods

Biodistribution of FITC-NZX in mice.

The ICR mice were intravenously injected with 20 mg/kg free FITC and FITC-labeled NZX. Mice was sacrificed and organ tissues (heart, liver, spleen, kidney and lung) were collected at 0.083 h, 2 h, 4 h, 6 h, 8 h and 12 h, respectively. The real-time fluorescence of organ tissues was observed by Maestro 2 IVIS Spectrum CT (PerkinElmer, USA) with the excitation wavelength at 500 nm and emission wavelength at 540 nm, respectively.

Results

Biodistribution of FITC-NZX in mice.

The biodistribution of NZX in heart, liver, spleen, kidney and lung was shown in Fig S1. The results showed that the fluorescence signal of FITC group was rapidly distributed in liver tissue, and disappeared in 2 h later. In addition, the fluorescence signal of FITC group was observed in spleen and lungs at 6 h, and disappeared later. However, the dynamic behavior of FITC-NZX group were different from FITC group. FITC-NZX mainly distributed in spleen, kidney and lung tissue, and fluorescence signal in the lung tissue last for 12 h, but the liver tissue had very weak fluorescence signal.

Figure S1. Biodistribution of FITC-NZX in mice. The ICR mice were injected intravenously with 20 mg/kg FITC (a negative control) **(A)** and FITC-labeled NZX **(B)**, and fluorescence intensity in organ tissues was observed at 0.083 h, 2 h, 4 h, 6 h, 8 h and 12 h, respectively. Organs tissues from left to right are liver, spleen, kidney, heart and lung, respectively.

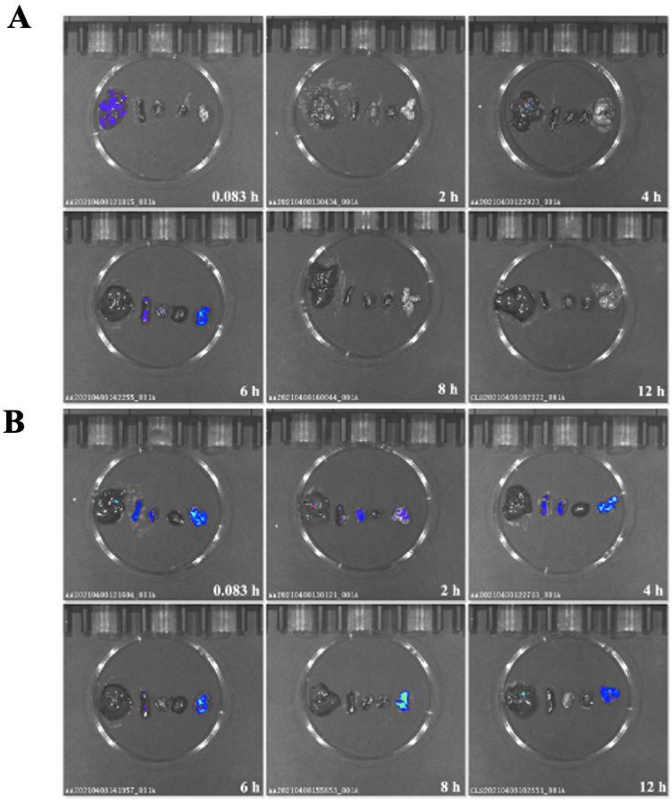

Fig. S1
